# Short BRAF+MEK coinhibition, alone and combined with anti‐CD20 immunotherapy, in relapsed or refractory hairy cell leukemia: An investigator‐sponsored multi‐center phase 2 clinical trial

**DOI:** 10.1002/hem3.70431

**Published:** 2026-07-09

**Authors:** Luca De Carolis, Monia Capponi, Alessandro Mancini, Flavio Falcinelli, Caterina Stelitano, Alessandro Pulsoni, Edoardo Simonetti, Alessandra Romano, Gianna Maria D'Elia, Jacopo Olivieri, Maria Paola Martelli, Alessandra Pucciarini, Alessia Santi, Marta Naccari, Alessandra Venanzi, Antonio Bennati, Pier Luigi Zinzani, Marzia Varettoni, Simone Ferrero, Massimo Pini, Robin Foà, Elisa Lucchini, Maurizio Frezzato, Brunangelo Falini, Enrico Tiacci

**Affiliations:** ^1^ Institute of Hematology, Department of Medicine and Surgery University and Hospital of Perugia Perugia Italy; ^2^ Hematology Hospital of Reggio Calabria Reggio Calabria Italy; ^3^ Hematology Sapienza University of Rome and Hospital of Latina Latina Italy; ^4^ Hematology University and Hospital of Catania Catania Italy; ^5^ Hematology Sapienza University and Hospital Umberto I of Rome Rome Italy; ^6^ Hematology Clinic, ASUFC Udine Italy; ^7^ IRCCS Azienda Ospedaliero‐Universitaria di Bologna Istituto di Ematologia “Seràgnoli” Bologna Italy; ^8^ Dipartimento di Scienze Mediche e Chirurgiche Università di Bologna Bologna Italy; ^9^ Hematology IRCCS S. Matteo Pavia Italy; ^10^ Hematology University of Turin and Hospital “Città della Salute e della Scienza” of Turin Turin Italy; ^11^ Hematology S. Giovanni Bosco Hospital Turin Italy; ^12^ Hematology Hospital of Trieste Trieste Italy; ^13^ Hematology Hospital of Vicenza Vicenza Italy

Hairy cell leukemia (HCL) is a rare chronic mature B‐cell neoplasm caused by the BRAF V600E kinase‐activating mutation in >95% of cases, and usually presents with cytopenia(s) and splenomegaly.^1^, ^2^, ^3^, ^4^ Although purine analogs (PNAs) ± rituximab yield frequent and durable remissions, up to 58% of patients relapse.^4^, ^5^, ^6^, ^7^ In phase 2 trials, monotherapy with the oral BRAF inhibitors vemurafenib and dabrafenib showed good activity when given for a few months to relapsed/refractory (R/R) HCL patients (30%–35% of complete remissions [CRs]).^8^, ^9^, ^10^, ^11^, ^12^ Still, minimal residual disease (MRD) persisted in the bone marrow (BM) even in CR patients, resulting in a median duration of response of up to 1.5 years overall. Interestingly, in our trial, we observed frequent ERK phosphorylation (pERK+) in residual BM HCL cells despite ongoing BRAF inhibition by vemurafenib. This finding correlated with a greater burden of leukemic residue and a shorter progression‐free survival (PFS),^8^ indicating MEK/ERK re‐activation bypassing BRAF as a potential resistance mechanism.

In a subsequent phase 2 trial of continuous BRAF+MEK coinhibition with dabrafenib+trametinib given for a median of ~3 years to R/R HCL patients, CRs were more frequent (66%), but MRD clearance was still rare (16%). Such continuous therapy sustained a high PFS at 2 years (94%), but led to frequent treatment‐related serious adverse events (SAEs; 35% of patients).^13^ Furthermore, therapeutic strategies of fixed and short duration would be desirable in a chronic disease like HCL that allows for an almost normal life expectancy in most cases.^14^, ^15^

In another phase 2 trial on R/R HCL, we showed that adding anti‐CD20 immunotherapy with rituximab (eight doses) to a short 8‐week course of vemurafenib yielded 87% CR, frequent MRD clearance (60%), and 78% PFS at ~3 years of median follow‐up.^16^ Such results were largely reproduced in a subsequent real‐life study of the same treatment regimen.^17^

Here, we tested in R/R HCL a short course of BRAF+MEK coinhibition with vemurafenib+cobimetinib first alone, and then combined with obinutuzumab (an anti‐CD20 agent potentially more active than rituximab^18^) in patients not responding to, or relapsing after, vemurafenib+cobimetinib.

From 8/2017 to 6/2019, 19 patients with R/R HCL needing treatment were enrolled across 7 Italian centers in a phase 2 investigator‐sponsored trial (EudraCT‐2017‐001836‐20) of 2–3 cycles (cycle = 28 days) of vemurafenib (960 mg b.i.d.; Days 1–28 of each cycle) and cobimetinib (60 mg daily; Days 1–21 of each cycle). Cycle‐3 was delivered only if no CR was obtained after cycle 2. Patient not responding to, or relapsing after, vemurafenib+cobimetinib, could receive up to three additional cycles of vemurafenib+cobimetinib combined with obinutuzumab (1000 mg for 8 doses over 6 cycles, starting with vemurafenib+cobimetinib). Inclusion criteria, response definitions, and assessment schedules are detailed in the Supplement.

A Simon's minimax two‐stage statistical design was used to target a CR rate of at least 45% at the end of vemurafenib+cobimetinib treatment in a per‐protocol analysis (primary endpoint), with *α* = 0.05 and *β* = 0.2.

Toxicities were graded according to CTCAEv4.03. If vemurafenib or cobimetinib dosing was to be suspended due to an adverse event attributable to one of these two drugs, per protocol also the other drug was to be held, and both drugs were to be concomitantly resumed for the total number of days planned, to ensure concurrent BRAF and MEK coinhibition for as much time as possible.

Patients (median age 58 years) had received a median of 3 prior therapies (Table 1), including 7/19 patients (37%) refractory to PNA and 4/19 (21%) exposed to a BRAF inhibitor alone or with rituximab. Median baseline neutrophils, hemoglobin, and platelets were 630/mm^3^, 10.4 g/dL, and 56,000/mm^3^, respectively.

**Table 1:** Patients characteristics.

Patients characteristics	Patients (*n* = 19)
Male sex (%)	15/19 (79)
Median age (range)	58 (42–73)
Median of prior lines of therapies (range)	3 (0^a^−9)

Previous treatments	No. of patients (%)
Cladribine	14 (74)
Pentostatin	9 (47)
Interferon alpha	8 (42)
Rituximab	4 (21)
Vemurafenib	2 (11)
Dabrafenib	2 (11)
Cladribine+rituximab	2 (11)
Pentostatin+rituximab	2 (11)
Splenectomy	2 (11)
Vemurafenib+rituximab	1 (5)
Rituximab+bendamustine	1 (5)
Rituximab+oral fludarabine	1 (5)
Venetoclax	1 (5)
Proportion of patients refractory to purine analogs (PA)	
Refractory to first course of PA (primary refractory)	3 (16)
Responsive to first course of PA, but refractory to a second or later PA course	4 (21)

Baseline disease parameters	Median (range)
Hemoglobin, g/dL	10.4 (7.4–14.4)
Platelets/mm^3^	56,000 (9,000–94,000)
Absolute neutrophil count/mm^3^	630 (37–2150)
HCL infiltration in bone marrow biopsy (%)	60 (22.5–90)
Spleen diameter—in centimeters—by ecography^b^	15 (11.4–27)

Duration of treatment (in weeks)	Median	IQR	Range
Duration of vemurafenib+cobimetinib treatment	9	8–12	1.3–12

Adherence to vemurafenib planned dosing	Median	IQR	Range
Dose intensity^c^	91%	86%–100%	16%–100%
Dose density^d^			
Patients (*n* = 6) receiving vemurafenib for 2 cycles	58 days	56–60 days	56–133 days
Patients (*n* = 8) receiving vemurafenib for 3 cycles	94 days	89–107 days	84–151 days

Adherence to cobimetinib planned dosing	Median	IQR	Range
Dose intensity^e^	92%	81%–100%	59%–100%
Dose density^f^			
Patients (*n* = 8) receiving cobimetinib for 2 cycles	46 days	42–74 days	42–126 days
Patients (*n* = 10) receiving cobimetinib for 3 cycles	82 days	64–94 days	63–120 days

Time to resolution of cytopenias (in days)^g^	Median (range)^h^
Anemia (*n* = 9^i^ patients)	59 (29–213)
Thrombocytopenia (*n* = 16^j^ patients)	22 (8–90)
Neutropenia (*n* = 16^k^ patients)	30 (9–155)

Response to vemurafenib+cobimetinib	Patients (%)
Non‐evaluable patients	2^l^/19
Evaluable patients:	17/19
Complete response	13/17 (76)
MRD‐negative	1/13 (8)
MRD‐positive	12/13 (92)
Partial response	2/17 (12)
Minor response	2/17 (12)

Abbreviations: HCL, hairy cell leukemia; IQR, interquartile range; MRD, minimal residual disease.

^a^ 2/19 patients had a previously untreated HCL but were unfit for chemotherapy with purine analogs due, in one case, to end‐stage renal disease, and in the other case, to chronic bronchiolitis treated with steroids for a prolonged time, followed by detection of *Mucorales* spp. in a bronchoalveolar lavage.

^b^ Longest spleen diameter in the 16 evaluable patients (of the remaining 3 patients, 2 were splenectomized, and in 1 the spleen diameter was not calculated).

^c^ Relative to the dose originally planned (i.e., 960 mg twice daily for two or three cycles), which was set at 100%; calculated in 18/19 patients (excluding the patient who deceased soon after starting treatment).

^d^ No. of days needed to complete the planned treatment schedule (i.e., 56 days if two cycles, 84 days if three cycles); calculated in 14/18 patients (since the 4 patients who prematurely discontinued vemurafenib due to toxicity could not be properly evaluated for dose density).

^e^ Relative to the dose originally planned (i.e., 60 mg daily, Days 1–21 of each cycle, for two or three cycles), which was set at 100%; calculated in 18/19 patients (excluding the patient who deceased soon after starting treatment).

^f^ No. of days needed to complete the planned treatment schedule (i.e., 42 days if two cycles, 63 days if three cycles).

^g^ Hemoglobin ≥ 11 g/dL, platelets ≥ 100,000/mm^3^, and neutrophils ≥ 1500/mm^3^.

^h^ Calculated in 18 evaluable patients (i.e., excluding the patient who deceased soon after starting treatment).

^i^ 9/18 evaluable patients had hemoglobin ≥ 11 g/dL at baseline evaluation.

^j^ Calculated in 16/18 patients, since 2/18 did not reach platelets ≥ 100,000/mm^3^ after treatment (1 minor response; 1 CR with incomplete recovery of platelets, which remained stable around 90,000/mm^3^ for 2 years before death from an unrelated cause).

^k^ Calculated in 17/18 patients, since 1/18 had absolute neutrophil count ≥ 1500/mm^3^ at baseline.

^l^ One patient normalized the blood counts but had an inadequate bone marrow (BM) biopsy post‐treatment. Another patient, an otherwise healthy 67‐year‐old woman with deep baseline neutropenia (196/mm^3^) and monocytopenia (0/mm^3^), developed a drug‐unrelated sepsis 9 days after starting therapy and deceased of shock with multi‐organ failure 5 days later.

Two of 19 patients were not evaluable for response post‐treatment (Table 1): one normalized the blood counts but had an inadequate BM biopsy post‐treatment; another one, an otherwise healthy 67‐year‐old woman with deep baseline neutropenia (196/mm^3^) and monocytopenia (0/mm^3^), developed a drug‐unrelated sepsis 9 days after starting therapy and deceased of shock with multi‐organ failure 5 days later. Thirteen of the 17 evaluable patients achieved CR (76%; primary endpoint P‐value: 0.000007), after two cycles (*n* = 8 patients) or three cycles (*n* = 5 patients) of therapy; all CRs but one were MRD‐positive in the BM aspirate by BRAF V600E allele‐specific polymerase chain reaction (PCR) (sensitivity threshold: ≥0.05% mutant alleles^16^). Two patients achieved a partial remission (PR; for an overall response rate of 88%, i.e., 15/17 patients), and 2 obtained a minor response. Almost all patients previously refractory to chemotherapy or exposed to a BRAF inhibitor responded (Supplementary Results).

Platelets, neutrophils, and hemoglobin quickly recovered above 100,000/mm^3^, 1500/mm^3^, and 11 g/dL after a median of 22, 30, and 59 days, respectively. Interestingly, HCL cells persistently expressing pERK were detected in BM biopsies taken during and/or at the end of treatment of only 1/9 evaluable cases (11%), versus 6/13 evaluable patients (46%) in our trial with vemurafenib alone.^8^ On the contrary, such pharmacodynamic data were not collected in the study of the other BRAF+MEK inhibitor doublet, that is, dabrafenib+trametinib.^13^ Our finding suggests that adding a MEK inhibitor may counteract resistance to single BRAF inhibition occurring via alternative routes that bypass BRAF and/or may more effectively silence vertical signaling from BRAF to ERK.

Indeed, at a median follow‐up of 36 months, median PFS was 38.4 months, and a sizeable subset of patients (4/19; 21%) was progression‐free at >5 years. In contrast, vemurafenib monotherapy in similar R/R populations (median of 3 prior treatments)—although delivered for longer periods (median of ~4–6 months)—yielded fewer CRs (30%–35%) and a less durable effect (median PFS ≤ 1.5 years, with no patients reported to be progression‐free beyond 5 years).^8^, ^10^ PFS after vemurafenib+cobimetinib seemed to be influenced by the number of prior treatments in multivariable analysis (Supplementary Results).

Treatment‐related toxicities (Table S1) were as expected from each drug, generally of low grade and always reversible (Supplementary Results). However, vemurafenib was permanently discontinued in four patients due to Grade 3 (*n* = 3) or recurrent Grade 2 (*n* = 1) rash, after 14–16.5 days of therapy. All four patients, after suspending both drugs per protocol, were able to resume cobimetinib monotherapy and achieved CR (*n* = 3) or PR (*n* = 1). No patients permanently discontinued cobimetinib. Temporary drug suspension and/or dose reduction often occurred, but median dose intensity was high for both vemurafenib (91%) and cobimetinib (92%), and dose density was good too (Table 1 and Supplementary Results).

This short course (≤3 months) of vemurafenib+cobimetinib indirectly compares well with continuous dabrafenib+trametinib treatment for a median of ~3 years^13^ in terms of treatment‐related SAE (16% vs. 35%, respectively) and CR rate (76% vs. 66%, respectively), despite the inclusion of patients previously exposed to a BRAF inhibitor (21% vs. 0%, respectively). Although the approach of continuous dabrafenib+trametinib treatment for years obviously sustained a high PFS (94% at 24 months,^13^ vs. a median PFS reached at 38.4 months with just a few months of vemurafenib+cobimetinib), benefits of fixed‐duration short treatment strategies include lower toxicity (clinical and financial), and opportunity for rechallenge at relapse^8^, ^9^, ^10^, ^16^, ^17^, ^19^ as the continuous selection pressure on the leukemic clone to acquire drug resistance is avoided.^20^ This likely explains the response to vemurafenib+cobimetinib we observed also in patients previously treated with fixed‐duration BRAF‐inhibitor therapy.

Indeed, patients were scheduled to receive on trial a second course of vemurafenib+cobimetinib with the addition of obinutuzumab if they did not respond to (*n* = 2 patients), or relapsed after (*n* = 10), the first course of vemurafenib+cobimetinib. Of these 12 eligible patients, 3 did not receive the second course for various reasons (Supplementary Results). The remaining 9 patients (median age: 59 years; median prior therapies: 4) received two cycles (*n* = 7) or three cycles (*n* = 2) of vemurafenib+cobimetinib together with obinutuzumab after a median of 41 months (range 1–79) following vemurafenib+cobimetinib. Toxicity was again manageable and mostly Grades 1–2, with only one treatment‐related SAE (Supplementary Results).

Notably, all 9 patients achieved CR with MRD‐negativity (100%), as compared to 7/9 CR (78%) with previous vemurafenib+cobimetinib, all of which MRD‐positive. This result cannot be explained only by obinutuzumab (which yielded 48% CRs in a trial on 26 R/R HCL patients with a median of 2 prior therapies^18^), and likely reflects also preserved disease sensitivity to MAPK pathway inhibition after a long treatment‐free interval in most patients. Responses to vemurafenib+cobimetinib+obinutuzumab were also durable, with 100% PFS at a median follow‐up of 33 months, versus a median PFS reached at 35.9 months in the same 9 patients after vemurafenib+cobimetinib (P‐value 0.054; Figure 1B). The vemurafenib+cobimetinib+obinutuzumab regimen compares indirectly well also with what is possibly the most effective chemotherapy‐free treatment regimen in the R/R HCL setting,^7^, ^21^, ^22^, ^23^ that is, vemurafenib+rituximab, which yielded 78% of PFS at 37 months in 30 patients with a median of 3 prior therapies who were largely naïve to BRAF inhibitors (23/30, 77%).^16^ Whether a MEK inhibitor adds value to a combination regimen including a BRAF inhibitor plus an anti‐CD20 monoclonal antibody and, if so, whether in the R/R setting triplet drug therapy should be delivered right away to all patients or should be reserved to a later use in case of no response or relapse after doublet drug therapy, are all questions newly raised by the current study that can be answered only by subsequent, ad hoc designed clinical trials.

**Figure 1:**
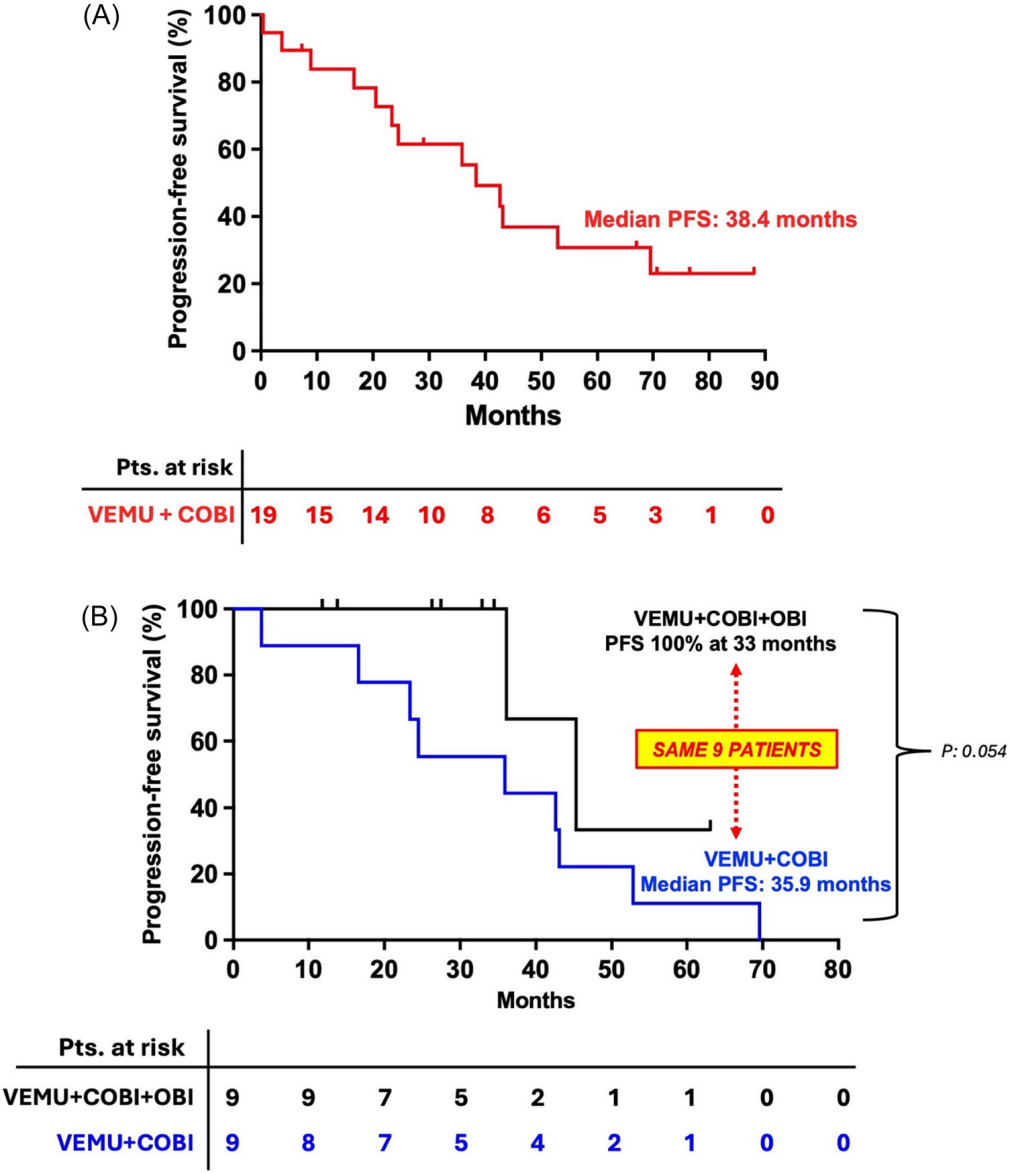
**(A)** Progression‐free survival (PFS) curve in patients treated with vemurafenib plus cobimetinib. **(B)** PFS after triplet therapy with vemurafenib+cobimetinib+obinutuzumab (black curve) was overall longer compared to the previous doublet therapy with vemurafenib+cobimetinib (blue curve) in the same nine patients sequentially receiving such regimens. Consistently, when comparing within individual patients the PFS after triplet versus doublet treatment, in 4/9 patients the response to the triplet treatment was more durable than that after initial doublet treatment, while the remaining 5/9 patients are still progression‐free after triplet treatment, but their current follow‐up is not informative in this regard, being shorter than the duration of the respective response after initial doublet treatment. COBI, cobimetinib; OBI, obinutuzumab; VEMU, vemurafenib.

A main limitation of this study is the relatively small number of patients enrolled. However, HCL is a rare neoplasm responding very well to first‐line chemotherapy, such that patients with relapsed or refractory HCL are even rarer. Despite the difficulty in performing investigator‐sponsored multi‐center clinical trials of targeted therapies in a rare disease setting of little or no commercial interest to pharmaceutical companies, we were able to show, in a reasonably sized population of 19 R/R patients, that a brief course of BRAF+MEK coinhibition with vemurafenib+cobimetinib is considerably active in R/R HCL. Moreover, in case of no response or relapse, addition of obinutuzumab to vemurafenib+cobimetinib re‐treatment produces even deeper and longer responses, with manageable toxicity and favorable efficacy compared to other therapeutic strategies in this setting.

## AUTHOR CONTRIBUTIONS

**Luca De Carolis**: Investigation; writing—review and editing. **Monia Capponi**: Investigation; writing—review and editing. **Alessandro Mancini**: Investigation; writing—original draft; data curation; formal analysis; writing—review and editing. **Flavio Falcinelli**: Investigation; writing—review and editing. **Caterina Stelitano**: Investigation; writing—review and editing. **Alessandro Pulsoni**: Investigation; writing—review and editing. **Edoardo Simonetti**: Investigation; writing—review and editing; data curation. **Alessandra Romano**: Investigation; writing—review and editing. **Gianna Maria D'Elia**: Investigation; writing—review and editing. **Jacopo Olivieri**: Investigation; writing—review and editing. **Maria Paola Martelli**: Investigation; writing—review and editing. **Alessandra Pucciarini**: Investigation; writing—review and editing. **Alessia Santi**: Investigation; writing—review and editing. **Marta Naccari**: Investigation; writing—review and editing. **Alessandra Venanzi**: Investigation; writing—review and editing. **Antonio Bennati**: Data curation; writing—review and editing. **Pier Luigi Zinzani**: Investigation; writing—review and editing. **Marzia Varettoni**: Investigation; writing—review and editing. **Simone Ferrero**: Investigation; writing—review and editing. **Massimo Pini**: Investigation; writing—review and editing. **Robin Foà**: Investigation; writing—review and editing. **Elisa Lucchini**: Investigation; writing—review and editing. **Maurizio Frezzato**: Investigation; writing—review and editing. **Brunangelo Falini**: Writing—review and editing; conceptualization; resources; project administration; funding acquisition; supervision; methodology; investigation. **Enrico Tiacci**: Conceptualization; methodology; data curation; formal analysis; supervision; funding acquisition; project administration; resources; writing—original draft; writing—review and editing; investigation.

## CONFLICT OF INTEREST STATEMENT

A.R.: research support from Pfizer.

P.L.Z.: consultant for MSD, AstraZeneca, and Novartis; speakers bureau for Recordati, Kite Gilead, Jannsen‐Cilag, BMS, SOBI, MSD, Regeneron, Takeda, Roche, AstraZeneca, Kyowa Kirin, Novartis, Incyte, BE ONE Medicines, and Eli Lilly; advisory board for Secura Bio, Recordati, Kite Gilead, Jannsen‐Cilag, BMS, SOBI, Sandoz, MSD, Regeneron, Takeda, Roche, AstraZeneca, Kyowa Kirin, Novartis, ADC Therapeutics, Incyte, BE ONE Medicines, and Eli Lilly.

S.F.: consultant for Janssen, EUSA Pharma, AbbVie, and Sandoz; component of the advisory board of Janssen, EUSA Pharma, Recordati, Incyte, Roche, AstraZeneca, Italfarmaco, and Behring; received speaker's honoraria from Janssen, EUSA Pharma, Recordati, Lilly, Beigene, Gilead, and Gentili; and received research funding from Gilead and Morphosys.

## FUNDING

This work was supported by the Leukemia and Lymphoma Society in conjunction with the Hairy Cell Leukemia (HCL) Foundation (grant no. 6557‐18 to E.T.), by the HCL Foundation in conjunction with the Sass Foundation for Medical Research (2016 grant program, to E.T. and B.F.), by Fondazione AIRC (grant IG‐19143 to E.T.; grant Metastasis/5‐per‐mille no. 21198 to R.F. and B.F.), and by the Italian Ministry of Health (grant RF‐2016‐02362264 to E.T.). We also acknowledge the support from Roche, which provided an unconditional research grant. Open access publishing facilitated by Università degli Studi di Perugia, as part of the Wiley ‐ CRUI‐CARE agreement.

## Supporting information

Supporting Information.

## Data Availability

The data that support the findings of this study are available from the corresponding author upon reasonable request.
