# Supplementary material for: Short BRAF+MEK coinhibition, alone and combined with anti‐CD20 immunotherapy, in relapsed or refractory hairy cell leukemia: An investigator‐sponsored multi‐center phase 2 clinical trial
Source: Hemasphere. 2026 Jul 9;10(7):e70431. doi: 10.1002/hem3.70431 (PMC13348899; doi:10.1002/hem3.70431)
Supplement: Supplementary file 1 — Supporting Information. [file HEM3-10-e70431-s001.docx]

**SUPPLEMENTARY METHODS**

**Study population**

The main categories of HCL patients needing anti-leukemic treatment who were eligible for inclusion in this trial were the following:

- Patients whose disease was refractory to therapy with purine analogues (no CR nor partial remission/PR, or relapse ≤1 year following treatment with cladribine or pentostatin).
- Patients who relapsed early (≥1 year and ≤2 years) after the first course of a purine analogue, or who relapsed whenever after a second or later course.
- Patients previously treated with a BRAF inhibitor (e.g., vemurafenib or dabrafenib).
- Patients who had manifested severe side effects from a previous therapy with purine analogues or were deemed by the investigator medically unfit for chemotherapy with purine analogues.

Furthermore, patients had to meet the following main inclusion/exclusion criteria:

- Male or female HCL patients ≥ 18 years of age.
- Proven diagnosis of HCL according to the morphological and immunophenotypic criteria (co-expression of CD11c/CD25/CD103 and/or positivity for annexin-A1), accompanied by the presence of the BRAF-V600E mutation
- ECOG performance status 0-2.
- Negative serum pregnancy test within 14 days prior to commencement of dosing in premenopausal women.
- Clinical indication for treatment, i.e. the presence of one or more of the following: neutrophils <1.5x10^9^ per liter, hemoglobin <11 g per deciliter, platelets <100x10^9^ per liter, bulky and/or symptomatic splenomegaly, clinically relevant infiltration of other organs (e.g., lymphadenopathy), recurrent disease-related opportunistic infections.

**Scheduled assessments for efficacy and safety**

During vemurafenib and cobimetinib dosing, visits and blood tests were performed weekly for the first cycle, then every other week for the second and, if applicable, the third cycle. Visits and blood test were also performed at every infusion of obinutuzumab, if applicable.

Bone marrow evaluation for response assessment at the end of treatment was performed the day after the last cycle (i.e., cycle 2 or cycle 3) in patients treated with vemurafenib + cobimetinib; and 4 weeks after the last obinutuzumab dose in patients subsequently treated with vemurafenib + cobimetinib + obinutuzumab. In patients treated with vemurafenib + cobimetinib, bone marrow evaluations were additionally scheduled after cycle 1, and pharmacodynamic assessment of persistent ERK phosphorylation in HCL cells during and/or the end of treatment was performed by immunohistochemistry as previously described^1^.

During follow-up post-treatment, visits and blood tests were performed every 3 months for the first two years; every 6 months for the following three years; and then annually.

**Definitions of response and progression**

A complete response (CR) required the blood counts to recover above the following thresholds without growth factor or transfusion support: neutrophils ≥1,500/mm^3^, hemoglobin ≥11 g/dl and platelets ≥100,000/mm^3^. Physical examination and/or imaging had to demonstrate disappearance of splenomegaly (and lymphadenopathy, if present). Nonimmunologic stains had to show the absence of HCL cells in the bone marrow biopsy.

CR without minimal residual disease (MRD) required also the absence of the BRAF-V600E mutation in the bone marrow and blood at allele-specific PCR (sensitivity threshold: 0.05% mutant alleles, as previously described^2^.

A partial response (PR) required the recovery of blood counts (as defined above for CR) and a reduction of ≥50% in the absolute counts of leukemic hairy cells in the bone marrow biopsy. On physical examination and/or imaging, a reduction of ≥50% of splenomegaly and lymphadenopathy (if present) was required.

An overall response (OR) was defined as either a CR or a PR.

A minor response (MR) was defined as an improvement of ≥50% in one or more abnormally low blood counts, and/or a reduction of ≥25% but <50% in the absolute counts of leukemic hairy cells in the bone marrow biopsy, and/or a reduction of ≥25% but <50% in hepato-splenomegaly and/or lymphadenopathy on physical examination and imaging.

Patients that did not meet the criteria for CR, PR or MR were judged to belong to the “no response” category.

In patients achieving PR or CR, relapse was defined as the occurrence of one or more of the following HCL-related events requiring treatment: neutrophils <1,500/mm^3^, hemoglobin <11 g/dl, platelets <100,000/mm^3^, bulky and/or symptomatic splenomegaly, recurrent disease-related opportunistic infections.

Progression was defined as: i) disease-related and clinically significant worsening of one or more blood counts and/or of splenomegaly; and/or ii) disease-related new occurrence or worsening of opportunistic infections; and/or iii) the death due to the disease.

Progression-free survival (PFS) was calculated from start of treatment until the date of progression. Overall survival (OS) was calculated from start of treatment until the date of death from any cause. Estimation of survival duration was assessed with the use of the Kaplan–Meier method, and statistical comparison of survival curves was performed through the Gehan-Breslow-Wilcoxon test. Patients had their data censored when they were found to be free from the event at the last available follow-up.

**SUPPLEMENTARY RESULTS**

**Response to vemurafenib and cobimetinib in patients previously refractory to chemotherapy or exposed to BRAF inhibitors**

All patients refractory to chemotherapy with a purine analog (n=7) had an OR after vemurafenib+cobimetinib, including 5/7 CR. Likewise, of the 4 patients previously exposed to a BRAF inhibitor 2 reached a CR; 1 had a durable resolution of cytopenias (for 38 months) without an evaluable post-treatment BM biopsy to discriminate between CR and PR; and a MR was obtained by the last patient, who had previously received not only a monotherapy with a BRAF inhibitor but also (upon relapse) a subsequent course of vemurafenib + rituximab.

**Toxicity of vemurafenib and cobimetinib**

Treatment-related toxicities (Table S1) were as expected from each drug, mostly of low grade, always reversible, and mainly represented by cutaneous rash and liver and pancreatic enzymes increase due to either drug; arthralgia and photosensitivity due to vemurafenib; and diarrhea, serous retinopathy and creatine phosphokinase increase due to cobimetinib. Treatment-related SAE were recorded in 3/19 patients (16%) and included cutaneous rash (n=2, both grade-3) and pancreatitis (n=1, grade-2).

**Temporary suspension and/or dose reductions of vemurafenib and cobimetinib**

Temporary suspension and/or dose reduction of vemurafenib and cobimetinib often occurred, but the drug dose could be successfully re-escalated in most patients.

In particular, among the 14 patients who did not discontinue vemurafenib permanently, vemurafenib was briefly interrupted in 9 cases (64%) for a median of 12 days (interquartile range [IQR] 6-35 days), and/or dose-reduced in 9 cases (64%; to a minimum of 720 mg b.i.d. in 6 cases and 480 mg b.i.d. in 3). Subsequent dose re‐escalation was feasible in most cases (6/9, including to 960 mg b.i.d. in 5 cases and 720 mg b.i.d. in 1 case), often with supportive low-dose corticosteroid therapy, underscoring the feasibility of proactive, steroid‐assisted toxicity management to target the planned vemurafenib exposure.

Cobimetinib was briefly interrupted in 11/18 patients (61%) for a median of 16.5 days (IQR 11-57 days), and/or dose‐reduced in 10 patients (56%; to a minimum of 40 mg/die in 4 cases, 20 mg/die in 3, 10 mg/die in 2, and 5 mg/die in 1), followed by dose re‐escalation in most cases (6/10, including to 60 mg/die in 3 cases and 40 mg/die in 3 cases).

**Multivariable analysis of progression-free survival (PFS) after treatment with vemurafenib and cobimetinib**

A post-hoc multivariate Cox proportional hazards regression analysis of PFS after vemurafenib+cobimetinib, including number of prior therapies and response to treatment (CR vs non-CR), showed, in the 17 patients with available data, higher risk of progression with increasing lines of treatments (hazard ratio/HR 1.617, 95% confidence interval/CI 1.15-2.41; p-value=0.0086), whereas response depth was not statistically significant (HR 2.02, 95% CI 0.26-10.7; p-value=0.4) possibly due to the low numbers of non-CR events (n=4).

**Patients eligible to, but not treated with, a second course of vemurafenib + cobimetinib with the addition of obinutuzumab**

Among the 12 eligible patients for a second course of vemurafenib+cobimetinib together with obinutuzumab due to cytopenia persistence (n=2) or recurrence (n=10), 3 patients were not treated for various reasons. One of the 2 patients persistently cytopenic had end-stage renal disease, advising against obinutuzumab due to its increased toxicity risk associated with kidney failure; still, despite reaching only a MR with vemurafenib + cobimetinib, he enjoyed a long PFS (71+ months) as his low-grade thrombocytopenia (~60,000/mm^3^) remained stable over time with no need for further treatment until death for causes unrelated to HCL. Among the 10 relapsed patients, one withdrew the consent and another one (with a previous history of allergy to both rituximab and obinutuzumab) had a clinically relevant infusion-related reaction to the obinutuzumab dose test (which was then permanently discontinued) and therefore received only vemurafenib+cobimetinib with significant clinical benefit (including an improvement in blood counts, although not meeting PR criteria), which is still maintained after 4.5 years through continuous therapy with these two drugs.

**Toxicity of vemurafenib + cobimetinib + obinutuzumab**

Toxicity of the triplet regimen was manageable, mostly grade 1-2, with no unexpected safety signals from adding obinutuzumab to vemurafenib+cobimetinib. Clinically relevant (but transient) grade ≥3 toxicities were one grade-4 thrombocytopenia requiring platelet transfusion due to obinutuzumab, and one mild edematous acute pancreatitis due to vemurafenib (grade-3 owing to the institution of medical therapy with pantoprazole; this was the only treatment-related SAE observed in the 9 patients, i.e. 11%).

**REFERENCES**

1. Tiacci, E. *et al.* Targeting Mutant BRAF in Relapsed or Refractory Hairy-Cell Leukemia. *New England Journal of Medicine* **373**, 1733–1747 (2015).

2. Tiacci, E. *et al.* Vemurafenib plus Rituximab in Refractory or Relapsed Hairy-Cell Leukemia. *New England Journal of Medicine* **384**, 1810–1823 (2021).

**SUPPLEMENTARY TABLE**

Table S1. Treatment-related adverse events during therapy with vemurafenib + cobimetinib in the 19 patients enrolled

| ***Adverse Drug reaction*** | ***No. of patients (%)*** | | | |
| --- | --- | --- | --- | --- |
|  | ***Grade 1-2*** | ***Grade 3*** | ***Grade 4*** | ***Total*** |
| Cutaneous rash/urticaria/itch | 11 (58%) | 3*(16%) | - | 14 (74%) |
| Diarrhea | 11 (58%) | - | - | 11 (58%) |
| Hyperbilirubinemia | 9 (47%) | 2 (11%) | - | 11 (58%) |
| ALP/GGT increase | 9 (47%) | 1 (5%) | - | 10 (53%) |
| Pancreatic enzyme increased° | 1 (5%) | 7 (37%) | 1 (5%) | 9 (47%) |
| Serous retinopathy/floaters | 8^(42%) | - | - | 8 (42%) |
| Arthralgia/arthritis | 8 (42%) | - | - | 8 (42%) |
| Creatine phosphokinase increase | 7 (37%) | 1 (5%) | - | 8 (42%) |
| AST/ALT increase | 6 (32%) | 1 (5%) | - | 7 (37%) |
| Photosensitivity reaction | 6 (32%) | - | - | 6 (32%) |
| QTc prolongation | 5 (26%) | - | - | 5 (26%) |
| Nausea/dyspepsia | 5 (26%) | - | - | 5 (26%) |
| Fatigue/malaise | 3 (16%) | - | - | 3 (16%) |
| Anemia | 2^†^ (11%) | - | - | 2 (11%) |
| Cutaneous neoplasms | 2^‡^ (11%) | - | - | 2 (11%) |
| Warts/melanocytic benign lesions | 2 (11%) | - | - | 2 (11%) |
| Fever | 2 (11%) | - | - | 2 (11%) |
| Rhabdomyolysis | 2 (11%) | - | - | 2 (11%) |
| Oral pain (aphthous stomatitis) | 2 (11%) | - | - | 2 (11%) |
| Pancreatitis | 1 (5%) | - | - | 1 (5%) |
| Muscle weakness | 1 (5%) | - | - | 1 (5%) |
| Lip infection (herpes labialis) | 1 (5%) | - | - | 1 (5%) |
| Uveitis | 1 (5%) | - | - | 1 (5%) |
| Myalgia | 1 (5%) | - | - | 1 (5%) |
| Blurred vision | 1 (5%) | - | - | 1 (5%) |
| Allergic reaction | 1 (5%) | - | - | 1 (5%) |
| eGFR worsening | - | 1^#^ (5%) | - | 1 (5%) |
| Abbreviation: eGFR, estimated Glomerular Filtration Rate  *Of these 3 rashes, all likely due to vemurafenib, 2 were deemed by the dermatologist as early Stevens-Johnson syndrome, although: i) no significant skin detachment was observed; ii) there was a prompt response to drug suspension and therapy with steroids; and iii) upon subsequent disease relapse, both patients were able to receive vemurafenib (in combination with cobimetinib and obinutuzumab) after de-sensitizing therapy with very low and gradually escalating vemurafenib doses.  °Lipase ± amylase elevation only (no clinical symptoms or radiological abnormalities) in all cases.  ^^^Only 2/8 patients (25%) had a grade-2 (symptomatic) event  ^†^Only one grade-2 event in a patient already having grade-1 anemia at baseline  ^‡^One basal cell carcinoma and one keratoacanthoma, both managed with simple excision  ^#^From grade-2 at baseline in a patient with pre-existing moderate to severe chronic kidney failure | | | | |
